# Fibrin glue as a stabilization strategy in peripheral nerve repair when using porous nerve guidance conduits

**DOI:** 10.1007/s10856-017-5889-4

**Published:** 2017-04-07

**Authors:** Divya Bhatnagar, Jared S. Bushman, N. Sanjeeva Murthy, Antonio Merolli, Hilton M. Kaplan, Joachim Kohn

**Affiliations:** 1 New Jersey Center for Biomaterials, Rutgers-The State University of New Jersey, 145 Bevier Road, Piscataway, NJ 08854 USA; 2 School of Pharmacy, University of Wyoming, 1000 E University Ave Dept. 3375, Laramie, WY 82071 USA

## Abstract

**Abstract:**

Porous conduits provide a protected pathway for nerve regeneration, while still allowing exchange of nutrients and wastes. However, pore sizes >30 µm may permit fibrous tissue infiltration into the conduit, which may impede axonal regeneration. Coating the conduit with Fibrin Glue (FG) is one option for controlling the conduit’s porosity. FG is extensively used in clinical peripheral nerve repair, as a tissue sealant, filler and drug-delivery matrix. Here, we compared the performance of FG to an alternative, hyaluronic acid (HA) as a coating for porous conduits, using uncoated porous conduits and reverse autografts as control groups. The uncoated conduit walls had pores with a diameter of 60 to 70 µm that were uniformly covered by either FG or HA coatings. In vitro, FG coatings degraded twice as fast as HA coatings. In vivo studies in a 1 cm rat sciatic nerve model showed FG coating resulted in poor axonal density (993 ± 854 #/mm^2^), negligible fascicular area (0.03 ± 0.04 mm^2^), minimal percent wet muscle mass recovery (16 ± 1 in gastrocnemius and 15 ± 5 in tibialis anterior) and G-ratio (0.73 ± 0.01). Histology of FG-coated conduits showed excessive fibrous tissue infiltration inside the lumen, and fibrin capsule formation around the conduit. Although FG has been shown to promote nerve regeneration in non-porous conduits, we found that as a coating for porous conduits in vivo, FG encourages scar tissue infiltration that impedes nerve regeneration. This is a significant finding considering the widespread use of FG in peripheral nerve repair.

**Graphical Abstract:**

## Introduction

Synthetic nerve conduits are often used to support peripheral nerve regeneration [1]. Mechanical properties such as flexibility and kink resistance, are important parameters while considering the design specifications of a synthetic conduit [2]. In addition, the porosity and permeability of a nerve conduit are also important [3]. The conduit walls can either be fabricated as non-porous or porous. Non-porous conduits work well when bridging smaller nerve gaps (<1 cm) while porous nerve conduits are particularly useful for long nerve gap repair because they allow diffusion of nutrients into the site of regeneration before the tube becomes vascularized [4–6].

It is known that the conduit wall porosity can impact nerve re-growth [7, 8]. Numerous studies have investigated conduit porosity in the context of peripheral nerve regeneration and reported contradictory results [9–11]. Increased porosity in the conduit walls may enhance permeability to nutrients, and this may potentially augment nerve regeneration [3]. However, larger pores (>30 µm) can also permit fibrous tissue infiltration and outward diffusion of growth factors from within the conduit lumen that can potentially suppress nerve regeneration [10, 12–15]. Therefore, optimizing the degree of conduit wall porosity is an essential step that requires the prevention of scar tissue infiltration while allowing inward diffusion of nutrients [16]. PLGA solution has been used to dip-coat braided PLGA nerve conduits in an attempt to reduce their pore size [17]. However, this attempt to reduce the conduit macropores failed to improve nerve regeneration. Clements et al. applied electrospun fiber mats made from tyrosine-derived polycarbonates, with hyaluronic acid (HA) hydrogel coating, to the conduit walls to reduce the pore size of braided conduits. They demonstrated that only the HA hydrogel barrier coatings, but not the electrospun fiber coatings, were effective in controlling the pore size of the conduits leading to improved functional nerve recovery in vivo [8].

Fibrin Glue (FG) has been used clinically as a tissue sealant in peripheral nerve repair for over four decades [18–22]. Reported benefits include decreased operative time [20], maintenance of correct fascicular orientation [23], improved gross surgical results [20], and minimal neural scarring with reduced fibrosis and inflammation [24]. It is also used widely experimentally, e.g., for stabilizing nerve repairs and for delivering neurotrophic factors [25–29]. In a recent in vivo mouse model study, FG sealant was found to reduce surgical repair time, enhance axonal regeneration and functional recovery [30]. Despite these benefits, it has been reported that there is some hesitancy to using it, due to fibrin’s role in increasing scar formation and hence potentially inhibiting nerve regeneration [23].

In addition to being used for nerve coaptation, FG has also been used to stabilize non-porous nerve conduits placed around the site of primary neurorhaphy to avoid additional suturing. This use of FG as an adjunct to conventional neuro-suturing resulted in better fiber alignment and axonal regeneration [31]. FG has also been used as a conduit material [32–36]. In addition, FG has been used as a filler within the lumen of non-porous conduits [37, 38], and as scaffolds to deliver growth factors for nerve regeneration [39, 40]. To the best of our knowledge, all the studies that used Fibrin Glue were done with non-porous conduits up till now. Although, FG is considered to be useful in nerve repair surgery, its utility in conjunction with porous conduits has not been investigated. In this study, we investigated the effectiveness of clinically used FG as a hydrogel coating for braided porous nerve conduits and compared it to the effectiveness of crosslinked HA coatings, uncoated porous conduits, and autograft repairs.

## Materials and methods

### Fabrication of braided conduits

The braided nerve conduits were fabricated using a tyrosine-derived polycarbonate (TyrPC) abbreviated as E1001(1k). This is a terpolymer composed of 10 mol% desaminotyrosyl tyrosine (DT), and 1 mol% poly(ethylene glycol) (PEG) of molecular weight 1 kDa; the remainder of 89 mol% is desaminotyrosyl tyrosine ethyl ester (DTE). A polymer with a weight average molecular weight of 333 kDa was synthesized using previously published procedures [41]. Briefly, the DTE and desaminotyrosyl-tyrosine *tert*-butyl ester (DTtBu) were copolymerized with a pre-determined molar equivalent of PEG under anhydrous conditions using bis(trichloromethyl) carbonate (triphosgene), followed by selective and quantitative removal of the *tert*-butyl ester protecting groups using trifluoroacetic acid (TFA) [42].

Braided conduits were fabricated using previously published procedures [8]. Briefly, E1001(1k) powder was melt-extruded into 80–110 µm diameter fibers on a Randcastle microextruder (Cedar Grove, NJ). The fibers were braided into a tubular conduit over a 1.5 mm diameter Teflon mandrel (Applied Plastics Co., Inc., Norwood, MA) using a tubular braiding machine (ATEX, Technologies Inc., Pinebuff, NC). The braiding parameters used were as follows: three filaments per yarn; 24 carriers, three twisted fibers/carrier, and traditional 2-over-2 braid. The conduits had an inner diameter of 1.5 mm, and the conduit walls had a uniform pore size distribution of about 65 ± 19 µm. The conduits were then cleaned by sonication in cyclohexane (1x), followed by 0.5% (v/v) Tween20 in DI water (1x) and lastly washing in DI water (5x). The cleaned conduits were vacuum dried overnight at room temperature. Finally, they were cut to 1.2 cm length with a thermal cutter and sterilized under UV light for 45 min.

### Hydrogel coatings

The braided conduits were coated with FG (Reagent Proteins, San Diego, CA) or HA (Glycosan Biosystems-BioTime, Inc. Alameda, CA). All the coating steps (Supplementary Fig. 1) were carried out aseptically. To coat with FG, the conduits were dipped once in a 1:1 mixture of fibrinogen (F) and thrombin (T) (150 µl of 50 Units/ml Thrombin and 150 µl of 50 mg/ml Fibrinogen) to achieve a ~200–250 μm thick coating. FG-coated conduits were dried overnight in a laminar flow hood before being used for implantation. To apply HA, conduits were coated by dipping in 1% (w/v) sterile thiol-modified hyaluronan solution for 30 s followed by crosslinking in 1% (w/v) poly(ethylene glycol diacrylate) (PEGDA) solution for 30 s. These steps were repeated 20 times to achieve a ~150–200 µm thick HA coating. After the 20th layer, the conduits were dipped in sterile hyaluronan solution for 30 s and then dried overnight in a laminar flow hood before being used for implantation. The optimized processing parameters for the HA and FG coating were selected after performing various iterations as shown in Supplementary Table 1.

### Scanning electron microscopy (SEM)

Conduits were imaged using scanning electron microscopy (SEM, Amray 1830I, 20 kV) after sputter coating with Au/Pd (SCD 004 sputter coater, 30 milliamps for 120 s). Pore size, thickness and uniformity of the coating were assessed on SEM images using Image J (software from National Institutes of Health). Coating thickness was calculated by subtracting the wall thickness of the uncoated conduit from the wall thickness of the coated conduit. Uniformity of the coating was assessed qualitatively. The coatings were considered to be ‘uniformly covering the pores’ when the pores between the 2-over-2 braids in the coated conduits were no longer visible in the SEM images.

### In vitro degradation of the hydrogel coatings

Degradation of the hydrogel coatings was followed over a 16-week period in vitro. Coated conduits (*n* = 3 per time point) were incubated in phosphate buffered saline (PBS; Life Technologies, NY, USA) at 37 °C for 1, 2, 4, 6, 8, 12 and 16 weeks. The coated conduits were air dried at room temperature and weighed over the 16-week study period. The percent weight loss of the hydrogel coatings was determined as $\left[ {\frac{{Wf - Wi}}{{Wi}}} \right]*100$, where *Wi* is the initial conduit weight prior to incubation, and *Wf* is the conduit weight at a particular time point. Dehydrated conduits at 1, 2, 4 and 6 weeks were also imaged using SEM to determine the integrity of the coatings.

### Mechanical characterization of the hydrogel coated conduits

Compression and three-point bending tests were performed to evaluate the mechanical properties of the braided hydrogel-coated conduits using a Syntec 5/D mechanical tester (MTS, Eden Prairie, MN). 1 and 1.5 cm long hydrogel coated conduits (*n* = 3 for each test) were incubated in PBS at 37 °C overnight and were tested immediately after removal from the incubator. The parameters used for the compression tests were: Conduit length = 1 cm, transverse crosshead speed = 6 mm/min; endpoint displacement = 60% of initial conduit diameter. Compressive structural stiffness was calculated from the slope of the linear region in the force vs. displacement curves. For 3-point bending tests, 1.5 cm long conduits (*n* = 3) were placed on the bending fixture over two roller pins that were 1 cm apart. The load was applied on a third pin located midway along the conduit at a crosshead speed of 10 mm/min. The slope determined by linear regression of the linear part of the force-displacement (F-d) curve was used to calculate bending stiffness. The results from three different coated conduits were averaged.

### In vivo evaluation

#### Surgical methods and groups

Nerve regeneration was evaluated in vivo in a 1 cm rat sciatic nerve injury model over a 16-week period. All experiments were conducted using protocols approved by the Rutgers Institutional Animal Care and Use Committee (IACUC). Twenty-four female Lewis rats (225–250 g) were randomly assigned to four groups: Uncoated, FG-coated, and HA-coated nerve conduits; and reverse autografts (*n* = 6 animals per group). The rats were anesthetized with isoflurane. The left sciatic nerve was exposed, and a 1 cm gap was created, 2 mm distal to the external obturator tendon. Sterile uncoated, HA-coated and FG-coated nerve conduits (1.2 cm long × 1.5 mm ID) were sutured to the distal and proximal nerve stumps using 9–0 nylon perineurial sutures (Ethicon, NJ, USA). For reverse autograft controls, the 1 cm nerve segment was reversed and sutured back into the same gap using 9–0 nylon sutures. The muscles and skin were closed using 6–0 Vicryl and nylon sutures respectively (Ethicon, NJ, USA). Skin sutures were removed 10 days after surgery.

#### Nerve conduction study

Peroneal and tibial electromyography (EMG) was performed to determine compound muscle action potential (CMAP) area and latency distal to the nerve injury (Viking Quest, CareFusion, San Diego, CA), as published previously [43]. The average of three consecutive recordings of the CMAP area and the latency were measured for each animal preoperatively, and then postoperatively at 4, 8, 12 and 16 weeks. The results were averaged for the animals in each treatment group.

#### Nerve histomorphometry and wet muscle mass

At the 16-week study endpoint, rats were anesthetized using isoflurane and the nerve was fixed in situ by filling the muscle pocket with Trump’s fixative (Electron Microscopy Sciences, Hatfield, PA) for 30 min. The tissue specimen (conduit/autograft including at least 3 mm of the proximal and distal nerve) were explanted and further fixed in Trump’s fixative for 4 days. The midsection of the conduit was post-fixed with osmium tetroxide, embedded in epoxy resin, cut into 1 μm thick sections and stained with toluidine blue. Images were acquired using a microscope at 50×, 400× and 1000× magnification and total myelinated area, axonal to fiber diameter (G-ratio), and axonal density for each group was calculated using Image J. After harvesting the nerve specimens, animals were sacrificed by CO_2_ asphyxiation and the tibialis anterior (TA) and gastrocnemius (Gastroc) muscles from both ipsilateral and contralateral sides of the hind leg were removed and immediately weighed to assess wet muscle mass.

## Results

### Conduit fabrication and hydrogel coatings

**Fig. 1:**
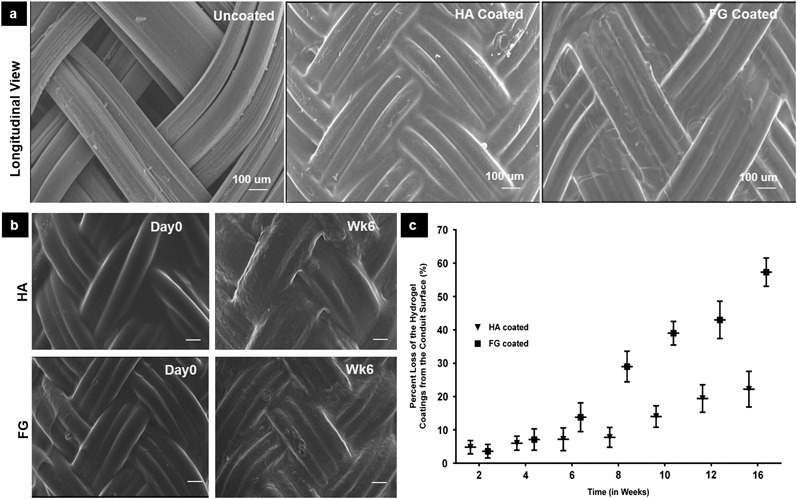
**a** SEM micrographs of the longitudinal view of the uncoated, HA and FG-coated conduits. An average pore size for uncoated conduits of 65 ± 19 µm was measured by Image J. After coating with either HA or FG, the coatings covered the macropores of the braided conduits, and no open pores were visible by SEM. [Scale bar = 100 µm]. **b** SEM micrographs of the HA and FG hydrogel coating after 6 weeks. The hydrogel coatings remain structurally intact at 6 weeks when compared to day 0. Scale bar = 100 µm **c** Weight (in percent of initial weight) of hydrogel coating remaining on the conduit surface after in vitro degradation over 16 weeks

The polymer identified as E1001(1k) was chosen from a library of tyrosine-derived polycarbonates to fabricate the braided porous conduits used in this study. The specific batch of E1001(1k) utilized in this study had a polydispersity (PDI) of 1.6, a glass transition temperature (T_g_) of 97 °C and a resorption period of ~18 months [44]. Extruded and oriented fibers with a diameter of about 80–110 µm were used to fabricate braided conduits using a 2-over-2 braid pattern. HA and FG hydrogels were prepared and applied as a coating on the outer side of the conduits. SEM micrographs (Fig. 1a) of the uncoated conduit show large open pores (65 ± 19 µm dia.) that were completely covered after coating. The coatings were considered to be uniform when no open pores could be visualized on SEM images. The average thickness of the HA coatings was ~160 ± 20 µm, and of the FG coatings was ~200 ± 25 µm.

### In vitro degradation and mechanical characterization of the hydrogel coated conduits

There was no noticeable degradation of the fibers in the conduit walls, which is expected given the 18 month resorption period of E1001(1k). SEM micrographs (Fig. 1b) showed that the HA and FG hydrogel coatings were structurally intact, and the macropores remain uniformly covered for 6 weeks in PBS (Fig. 1b). However, the FG coating degrades faster than the HA coating: at 16 weeks, there is >50% loss of the FG coating, compared to 20% loss of the HA coating **(**Fig. 1c **)**. Mechanical properties of the uncoated and coated braided conduits were also evaluated by transverse compressive load-displacement analysis and three-point bending tests. Our results (Table 1) show that the coated conduits have a slightly higher compressive structural stiffness, but similar bending stiffness as the uncoated conduits.

**Table 1:** Compressive structural stiffness and bending stiffness of the uncoated conduit compared to the HA-coated and FG-coated conduit (mean ± SD of the three samples tested)

	Uncoated conduit	HA coated conduit	FG-coated conduit
Compressive structural stiffness (N/mm)	23 ± 2	28 ± 3	30 ± 5
Bending stiffness (EI, Nmm^2^)	15 ± 5	15 ± 3	17 ± 3

### In vivo evaluation

#### Scar tissue response and histomorphometric analysis at 16 weeks

**Fig. 2:**
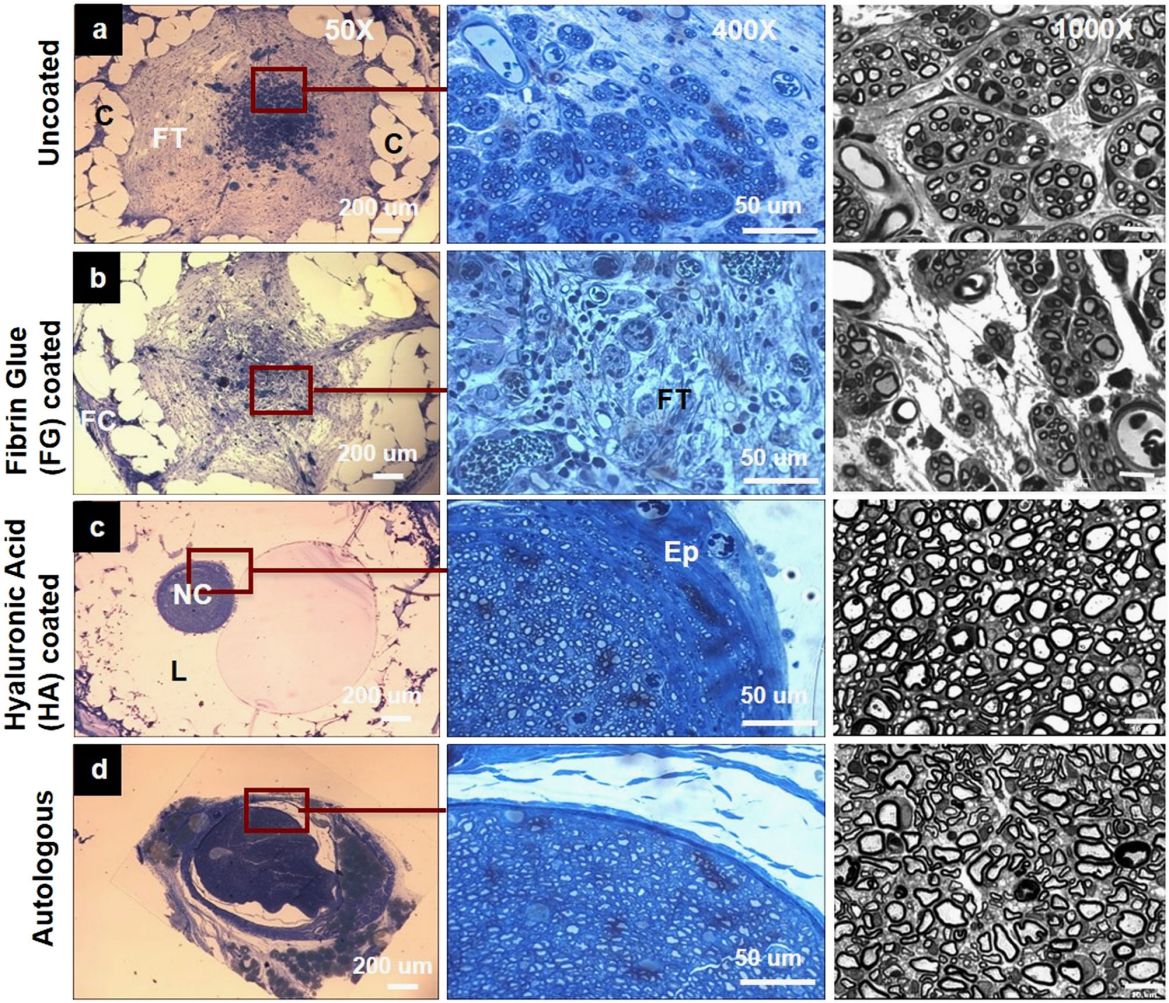
Toluidine blue stained 1 μm-thick cross sections of the conduits explanted after 16 weeks in vivo. 50× images show entire conduit and nerve cable (dark stain) [Scale bar = 200 µm], 400× images show the interface between axonal tissue and surrounding inter-luminal area [Scale bar = 50 µm] and 1000× images represent the 1 µm-thick nerve mid-segments post-fixed with osmium tetraoxide for **a** Uncoated **b** FG-coated (very few myelinated axons present in some cases) **c** HA-coated (high density of myelinated axons) **d** Autologous. [Scale bar = 10 µm]. (*FT* fibrous tissue, *Ep* Epineurium, *C* Conduit, *L* lumen, *FC* fibrous capsule, *NC* nerve cable)

After fixation, and embedding of the explanted conduits, they were sectioned into 1 µm-thick slices and stained with osmium tetroxide and toluidine blue. Histological evaluation of the HA-coated and FG-coated nerve conduit sections demonstrated a marked difference in tissue composition at 16 weeks (Fig. 2). The regenerated nerves within uncoated braided conduits were poorly organized (Fig. 2a). The nerve cable contained numerous myelinated axons, but these were dispersed, instead of constituting well-defined fascicles. Myelinated axons were surrounded by fibrous tissue (denoted by FT in the figure) that appeared to have impeded the formation of a well-defined nerve cable surrounded by epineurium.

Although the FG and HA hydrogel coatings cover the pores of braided conduits in a similar manner in vitro (SEM micrographs, Fig. 1a), they induce different types of cellular infiltration in vivo. FG coating on the E1001(1k) conduits not only failed to prevent the infiltration of scar tissue but appeared to enhance it (Fig. 2b). There was no observable axonal regeneration in FG-coated conduits. Figure 2b demonstrates a thick capsule of scar tissue around the FG-coated conduit.

In contrast to FG, the HA coating prevented the infiltration of non-nerve tissue and led to the formation of a densely packed nerve cable with a high density of myelinated axons (Fig. 2c). The nerve cable was surrounded by a well-defined epineurium, characteristic of healthy regeneration, and similar to that seen with autograft. Autografts (Fig. 2d) indeed showed numerous myelinated axons in a well-defined epineurium.

Histomorphometric analysis was performed using 1000× images to compare the morphology of the regenerating axons and the myelin sheath. Total surface area of the axonal tissue, the density of myelinated nerve fibers and the G-ratio were calculated using Image J. FG-coated conduits had the lowest axonal density while HA-coated conduits and autografts had a very high axonal density (Table 2). FG-coated conduits had the lowest fascicular area due to a low density of myelinated axons compared to autografts and HA coated nerve conduits. G-ratio (axon/fiber diameter) was highest in the FG-coated conduits, indicating less myelination. G-ratio of the autografts and HA-coated conduits were close to the normal value of healthy nerves (0.6), an optimal value for the transmission of current from one node of Ranvier to the next [45].

**Table 2:** Nerve histomorphometry of the four comparative groups: Average density of the myelinated nerve fibers: Axonal Density (#/mm^2^); The total surface area of the axonal tissue: Fascicular area (mm^2^); Mean G-ratio: the ratio of inner axonal diameter to the total outer diameter

Groups	Average axonal density (#/mm^2^)	Fascicular area (mm^2^)	G-ratio
Autologous	6460 ± 362	0.36 ± 0.08	0.64 ± 0.02
Uncoated	5382 ± 428	0.15 ± 0.02	0.69 ± 0.02
FG-coated	993 ± 854	0.03 ± 0.04	0.73 ± 0.01
HA-coated	7130 ± 306	0.17 ± 0.04	0.66 ± 0.02

Data are represented as mean ± SE

#### Wet muscle mass and functional recovery

The percent wet muscle mass recovery for gastrocnemius and tibialis anterior muscles was calculated as (Ipsilateral muscle mass/Contralateral muscle mass) *100 at 16 weeks (Table 3).

**Table 3:** Percent wet muscle mass recovery at the end of 16 weeks for Gastrocnemius and Tibialis Anterior muscles

Groups	Gastrocnemius muscle recovery (%)	Tibialis anterior muscle recovery (%)
Autologous	60 ± 6	69 ± 4
Uncoated	34 ± 3	42 ± 2
FG-coated	16 ± 1	15 ± 5
HA coated	50 ± 2	60 ± 3

Rats implanted with FG-coated conduits had the lowest muscle weight compared to highest muscle weight in autologous implants, followed by HA-coated conduits and then uncoated conduits.

**Fig. 3:**
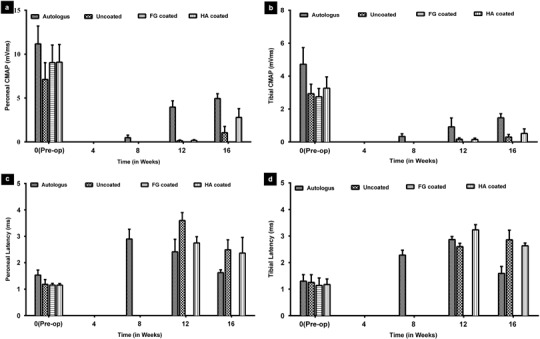
Electrophysiological measurements pre and post-op. every 4 weeks till 16 weeks. CMAP area **a** Peroneal **b** Tibial; CMAP Latency: **c** Peroneal and **d** Tibial nerve

To assess the extent of functional regeneration, we measured the peroneal and tibial CMAP area (Fig. 3a and b) and latency (Fig. 3c and d), pre-operatively and post-operatively over the 16-week period. Autografts had faster and more complete functional recovery than nerves that regenerate in conduits (Fig. 3a and b). In autografts, initial nerve conduction and CMAP area recovery was seen at week 8 as compared to week 12 for HA-coated conduits and uncoated conduits. There was no CMAP recovery for any of the FG-coated conduits, even at week 16 (Fig. 3a and b). This corroborates the histological findings described above for the FG-coated conduits (Fig. 2). Figure 3c and d shows normal latencies pre-operatively, which were never fully regained after injury. Distal latency for the autografts was detected at week 8 and for the HA conduit group and uncoated conduits at week 12. Even at 16 weeks, no latency could be measured for the FG-coated conduits.

## Discussion

Fibrin Glue is one of the most extensively used materials in peripheral nerve regeneration. Conduits fabricated from fibrin have been demonstrated to increase axonal growth compared to a poly-3-hydroxybutyrate (PHB) conduit [46], and to promote regeneration of up to 86% myelinated axons when compared to a nerve graft in a 1 cm rat sciatic nerve model [47]. As a filler and scaffold within the non-porous conduits, fibrin glue has been used for encapsulating and delivering mesenchymal stem cells in a non-porous poly(caprolactone) (PCL) conduit [48]. In another study, Ma et al. successfully used non-porous silicone tubes filled with nerve growth factor (NGF) - FG mixtures to repair a 1.5 cm rat sciatic nerve gap [49]. The most frequent use of fibrin glue has been as a tissue sealant for nerve coaptation [24]. Several studies have shown the potential benefits of using fibrin glue compared with microsuturing [50, 51]. Isaacs et al. showed that fibrin glue holds the nerve ends, maintaining the overall alignment and providing a barrier from intervening scar tissue [52]. Since fibrin glue is so widely and successfully used for non-porous nerve conduits in various forms, it encouraged us to explore its use for porous nerve conduits.

Braiding introduces porosity in the nerve conduit wall [8, 17, 53–55]. Gao et al. showed that braided nerve conduits made of filaments of poly(L-lactide-co-glycolide) (PLGA) were highly porous (pore size between 50–100 µm) [54]. The uncoated E1001(1k) braided nerve conduits used in this study also had large open pores (65 ± 19 µm dia.) (Fig. 1). Considering that non-neural cells can easily penetrate through the large pores of these braided nerve conduits, in this study, we investigated the use of fibrin glue as a hydrogel coating to cover these pores. FG uniformly covered the pores of the uncoated E1001(1k) nerve conduit and was intact in vitro until 6 weeks. However, by 16 weeks, FG coating had degraded >50%.

Mechanical properties of a nerve conduit are critical for the conduit to maintain its integrity, stability at the implant site [56] and to resist against the compression caused by the surrounding tissues, muscular contraction, and deformation [57]. Our mechanical tests suggested that the hydrogel coatings slightly increased the compressive structural stiffness of the conduits but the bending stiffness remained the same as for the uncoated porous conduits.

Histology of the regenerated nerves within the conduits strongly indicated that FG coating on the E1001(1k) conduits not only failed to prevent the infiltration of scar tissue but appeared to enhance it. Formation of a thicker capsule of tissue around the FG-coated conduits than the HA-coated ones suggests that the FG coating may be attracting the fibroblasts present in the conduit periphery. A similar fibrous capsule outside the conduit and fibrous tissue infiltration were also reported in bi-component electrospun conduits [43]. Fibrous tissue infiltration was also reported in commercial collagen nerve tubes, which failed to regenerate nerves [58]. Histology of the midsegment of their explanted collagen conduits confirmed the formation of fibrous scar tissue, which resulted in a disorganized neural architecture and very little axonal regeneration. During the period of nerve regeneration, axons begin to regenerate from the proximal towards the distal ends, and Schwann cells start to migrate into the conduit lumen [59]. The infiltration of fibrous tissue as seen in the uncoated conduits, and in conduits with FG coating, can impede neural regeneration, misdirect axons, and interfere with the maturation of sprouting axons [60]. Results from the histomorphometric analysis confirmed that FG coatings fail to prevent infiltration of non-nerve tissue.

Denervation of the target muscle is also an indication of peripheral nerve injury [61]. As the nerve regenerates into the muscle, the neuromuscular junction is restored, and the muscle regains its mass in proportion to the degree of reinnervation [62]. The lower degree of wet muscle mass recovery with FG-coated conduits indicated poorer nerve regeneration. This finding was also corroborated by the histological and the histomorphometric findings.

Electrophysiological findings over 16-weeks indicated decreased CMAP areas in the animals implanted with FG-coated conduits. CMAP area is proportional to the number of motor axons stimulated and hence, correlates with the number of regenerated axons [63]. As a complementary measure to amplitude, CMAP latencies are indicative of nerve conduction velocity, and correlate with the level of axonal myelination and nerve regeneration. Decreased latencies are indicative of faster conduction velocity and improved recovery [64]. Even at 16 weeks, no latency could be measured in animals with FG-coated conduits, indicating very little, if any, nerve regeneration. These electrophysiological recovery assessments are in line with wet muscle mass and histomorphometric results.

## Conclusions

Considering the wide use of FG as a tissue sealant in nerve repair, the results of our study are unexpected. FG performed poorly when used as a coating for porous braided conduits. Our results indicate that a FG coating on a porous conduit appears to attract non-neural cells. Instead of preventing the infiltration of scar tissue into the lumen of the conduit, the presence of a FG coating seemed to facilitate it. Consequently, FG coatings resulted in poor axonal regeneration and functional recovery. We conclude that although FG has been successfully used in conjunction with *non-porous* conduits to facilitate peripheral nerve regeneration, its use as a coating on *porous* conduits impedes functional nerve regeneration. In contrast, our results indicate that HA coatings are more effective than FG coatings in preventing scar tissue infiltration into the lumen of porous conduits.

## Electronic supplementary material

Supplementary Table 1

Supplementary Fig. 1

## References

[1] Mackinnon SE, Dellon AL (1990). Clinical nerve reconstruction with a bioabsorbable polyglycolic acid tube. Plast Reconstr Surg.

[2] Hudson TW, Evans G, Schmidt CE (1999). Engineering strategies for peripheral nerve repair. Clin Plast Surg.

[3] Arslantunali D, Dursun T, Yucel D, Hasirci N, Hasirci V (2014). Peripheral nerve conduits: technology update. Med Devices (Auckl).

[4] de Ruiter GC, Malessy MJ, Yaszemski MJ, Windebank AJ, Spinner RJ (2009). Designing ideal conduits for peripheral nerve repair. Neurosurg Focus.

[5] Li S-T, Yuen D. Implant devices for nerve repair. Google Patents. 2004.

[6] Griffith LG (2002). Emerging design principles in biomaterials and scaffolds for tissue engineering. Ann NY Acad Sci.

[7] Oh SH, Kim JH, Song KS, Jeon BH, Yoon JH, Seo TB (2008). Peripheral nerve regeneration within an asymmetrically porous PLGA/Pluronic F127 nerve guide conduit. Biomaterials.

[8] Clements BA, Bushman J, Murthy NS, Ezra M, Pastore CM, Kohn J (2016). Design of barrier coatings on kink-resistant peripheral nerve conduits. J Tissue Eng.

[9] Chamberlain L, Yannas I, Arrizabalaga A, Hsu H-P, Norregaard T, Spector M (1998). Early peripheral nerve healing in collagen and silicone tube implants: myofibroblasts and the cellular response. Biomaterials.

[10] Jenq C-B, Jenq LL, Coggeshall RE (1987). Nerve regeneration changes with filters of different pore size. Exp Neurol.

[11] Vleggeert-Lankamp CL, Wolfs J, Pêgo AP, Van Den Berg R, Feirabend H, Lakke E (2008). Effect of nerve graft porosity on the refractory period of regenerating nerve fibers. J Neurosurg.

[12] Oh SH, Kim JR, Kwon GB, Namgung U, Song KS, Lee JH (2013). Effect of surface pore structure of nerve guide conduit on peripheral nerve regeneration. Tissue Eng Part C Methods.

[13] Guenard V, Valentini RF, Aebischer P (1991). Influence of surface texture of polymeric sheets on peripheral nerve regeneration in a two-compartment guidance system. Biomaterials.

[14] Wang X, Cui T, Yan Y, Zhang R (2009). Peroneal nerve regeneration using a unique bilayer polyurethane-collagen guide conduit. J Bioact Compat Polym.

[15] Meek MF, Den Dunnen WF (2009). Porosity of the wall of a Neurolac® nerve conduit hampers nerve regeneration. Microsurgery.

[16] Kokai LE, Lin Y-C, Oyster NM, Marra KG (2009). Diffusion of soluble factors through degradable polymer nerve guides: controlling manufacturing parameters. Acta Biomater.

[17] Bini T. Development of novel microbraided scaffolds for nerve tissue engineering. National University of Singapore, Singapore. 2004.

[18] Wang LZ, Gorlin J, Michaud SE, Janmey PA, Goddeau RP, Kuuse R (2000). Purification of salmon clotting factors and their use as tissue sealants. Thromb Res.

[19] Choi BH, Han SG, Kim SH, Zhu SJ, Huh JY, Jung JH (2005). Autologous fibrin glue in peripheral nerve regeneration in vivo. Microsurgery.

[20] Narakas A (1988). The use of fibrin glue in repair of peripheral nerves. Orthop Clin North Am.

[21] Pierce NE, Alt JA, Antonelli PJ (2015). Hydrogel sutureless facial nerve repair: pilot clinical investigation. Laryngoscope.

[22] Grinsell D, Keating C. Peripheral nerve reconstruction after injury: a review of clinical and experimental therapies. BioMed Res Int. 2014;2014:1–13. 10.1155/2014/698256 PMC4167952 25276813

[23] Rafijah G, Bowen AJ, Dolores C, Vitali R, Mozaffar T, Gupta R (2013). The effects of adjuvant fibrin sealant on the surgical repair of segmental nerve defects in an animal model. J Hand Surg [Am].

[24] Sameem M, Wood TJ, Bain JR (2011). A systematic review on the use of fibrin glue for peripheral nerve repair. Plast Reconstr Surg.

[25] Huang JH, Cullen DK, Browne KD, Groff R, Zhang J, Pfister BJ (2009). Long-term survival and integration of transplanted engineered nervous tissue constructs promotes peripheral nerve regeneration. Tissue Eng Part A.

[26] Félix SP, Pereira Lopes FR, Marques SA, Martinez A (2013). Comparison between suture and fibrin glue on repair by direct coaptation or tubulization of injured mouse sciatic nerve. Microsurgery.

[27] Belkas JS, Shoichet MS, Midha R (2004). Peripheral nerve regeneration through guidance tubes. Neurol Res.

[28] Cheng H, Cao Y, Olson L (1996). Spinal cord repair in adult paraplegic rats: partia restoration of hind limb function. Science.

[29] Guest JD, Hesse D, Schnell L, Schwab ME, Bunge MB, Bunge RP (1997). Influence of IN‐1 antibody and acidic FGF‐fibrin glue on the response of injured corticospinal tract axons to human Schwann cell grafts. J Neurosci Res.

[30] Koulaxouzidis G, Reim G, Witzel C (2015). Fibrin glue repair leads to enhanced axonal elongation during early peripheral nerve regeneration in an in vivo mouse model. Neural Regen Res.

[31] Theberge NP, Ziccardi VB. Use of fibrin glue as an adjunct in the repair of lingual nerve injury: case report. J Oral and Maxillofac Surg. 2016;74(9):1899e1–4 10.1016/j.joms.2016.04.027 27235179

[32] Pabari A, Yang SY, Mosahebi A, Seifalian AM (2011). Recent advances in artificial nerve conduit design: strategies for the delivery of luminal fillers. J Control Release.

[33] Jin J, Limburg S, Joshi SK, Landman R, Park M, Zhang Q (2013). Peripheral nerve repair in rats using composite hydrogel-filled aligned nanofiber conduits with incorporated nerve growth factor. Tissue Eng Part A.

[34] di Summa PG, Kalbermatten DF, Pralong E, Raffoul W, Kingham PJ, Terenghi G (2011). Long-term in vivo regeneration of peripheral nerves through bioengineered nerve grafts. Neuroscience.

[35] Pettersson J, McGrath A, Kalbermatten DF, Novikova LN, Wiberg M, Kingham PJ (2011). Muscle recovery after repair of short and long peripheral nerve gaps using fibrin conduits. Neurosci Lett.

[36] Longo MVL, Marques de Faria JC, Isaac C, Nepomuceno AC, Teixeira NH, Gemperli R (2016). Comparisons of the results of peripheral nerve defect repair with fibrin conduit and autologous nerve graft: an experimental study in rats. Microsurgery.

[37] Nakayama K, Takakuda K, Koyama Y, Itoh S, Wang W, Mukai T (2007). Enhancement of peripheral nerve regeneration using bioabsorbable polymer tubes packed with fibrin gel. Artif Organs.

[38] Nakayama K, Takakuda K, Koyama Y, Itoh S, Wang W, Shirahama N (2007). Regeneration of peripheral nerves by bioabsorbable polymer tubes with fibrin gel. J Nanosci Nanotechnol.

[39] Tajdaran K, Gordon T, Wood MD, Shoichet MS, Borschel GH (2016). An engineered biocompatible drug delivery system enhances nerve regeneration after delayed repair. J Biomed Mater Res A.

[40] Reichenberger MA, Mueller W, Hartmann J, Diehm Y, Lass U, Koellensperger E et al. ADSCs in a fibrin matrix enhance nerve regeneration after epineural suturing in a rat model. Microsurgery. 2015;36(6):491–500. 10.1002/micr.30018 26716599

[41] Magno MHR, Kim J, Srinivasan A, McBride S, Bolikal D, Darr A (2010). Synthesis, degradation and biocompatibility of tyrosine-derived polycarbonate scaffolds. J Mater Chem.

[42] Magno MHR. Optimization of tyrosine-derived polycarbonate terpolymers for bone regeneration scaffolds: rutgers. Rutgers, The State University of New Jersey, Piscataway, New Jersey, USA; 2012.

[43] Cirillo V, Clements BA, Guarino V, Bushman J, Kohn J, Ambrosio L (2014). A comparison of the performance of mono-and bi-component electrospun conduits in a rat sciatic model. Biomaterials.

[44] Bhatnagar D, Dube K, Damodaran VB, Subramanian G, Aston K, Halperin F et al. Effects of terminal sterilization on PEG‐based bioresorbable polymers used in biomedical applications. Macromol Mater Eng. 2016;301(10):1211–1224 10.1002/mame.201600133 PMC5340269 28280451

[45] Kääriäinen M, Kauhanen S (2012). Skeletal muscle injury and repair: the effect of disuse and denervation on muscle and clinical relevance in pedicled and free muscle flaps. J Reconstr Microsurg.

[46] Kalbermatten DF, Pettersson J, Kingham PJ, Pierer G, Wiberg M, Terenghi G (2009). New fibrin conduit for peripheral nerve repair. J Reconstr Microsurg.

[47] Pettersson J, Kalbermatten D, McGrath A, Novikova LN (2010). Biodegradable fibrin conduit promotes long-term regeneration after peripheral nerve injury in adult rats. J Plast Reconstr Aesthet Surg.

[48] Cartarozzi LP, Spejo AB, Ferreira RS, Barraviera B, Duek E, Carvalho JL (2015). Mesenchymal stem cells engrafted in a fibrin scaffold stimulate Schwann cell reactivity and axonal regeneration following sciatic nerve tubulization. Brain Res Bull.

[49] Ma S, Peng C, Wu S, Wu D, Gao C (2013). Sciatic nerve regeneration using a nerve growth factor-containing fibrin glue membrane. Neural Regen Res.

[50] Martins RS, Siqueira MG, Da Silva CF, Plese JP (2005). Overall assessment of regeneration in peripheral nerve lesion repair using fibrin glue, suture, or a combination of the 2 techniques in a rat model. Which is the ideal choice?. Surg Neurol.

[51] Ornelas L, Padilla L, Di Silvio M, Schalch P, Esperante S, Infante RL (2006). Fibrin glue: an alternative technique for nerve coaptation-Part II. Nerve regeneration and histomorphometric assessment. J Reconstr Microsurg.

[52] Isaacs JE, McDaniel CO, Owen JR, Wayne JS (2008). Comparative analysis of biomechanical performance of available “nerve glues”. J Hand Surg [Am].

[53] Liu G, Hu H, Zhang P, Wang W (2005). An experimental investigation into the radial compressive properties of the biodegradable braided regeneration conduits for peripheral nerve repair. Autex Res J..

[54] Gao S, Wang S, Ramakrishna S (2005). Development of fibrous biodegradable polymer conduits for guided nerve regeneration. J Mater Sci: Mater Med.

[55] Bini T, Gao S, Xu X, Wang S, Ramakrishna S, Leong K (2004). Peripheral nerve regeneration by microbraided poly (L‐lactide‐co‐glycolide) biodegradable polymer fibers. J Biomed Mater Res A.

[56] Yao L, Billiar KL, Windebank AJ, Pandit A (2010). Multichanneled collagen conduits for peripheral nerve regeneration: design, fabrication, and characterization. Tissue Eng Part C: Methods.

[57] Topp KS, Boyd BS (2006). Structure and biomechanics of peripheral nerves: nerve responses to physical stresses and implications for physical therapist practice. Phys Ther.

[58] Moore AM, Kasukurthi R, Magill CK, Farhadi HF, Borschel GH, Mackinnon SE (2009). Limitations of conduits in peripheral nerve repairs. Hand.

[59] Schmidt CE, Leach JB (2003). Neural tissue engineering: strategies for repair and regeneration. Annu Rev Biomed Eng.

[60] Burnett MG, Zager EL (2004). Pathophysiology of peripheral nerve injury: a brief review. Neurosurg Focus.

[61] Feng X, Yuan W (2015). Dexamethasone enhanced functional recovery after sciatic nerve crush injury in rats. BioMed Res Int.

[62] Wang W, Itoh S, Matsuda A, Ichinose S, Shinomiya K, Hata Y (2008). Influences of mechanical properties and permeability on chitosan nano/microfiber mesh tubes as a scaffold for nerve regeneration. J Biomed Mater Res A.

[63] Smit X. Struggle at the site of nerve injury: a rat sciatic nerve study on fundamental problems of peripheral nerve injury. Erasmus Universiteit Rotterdam, Netherlands; 2006.

[64] Daly WT, Knight AM, Wang H, de Boer R, Giusti G, Dadsetan M (2013). Comparison and characterization of multiple biomaterial conduits for peripheral nerve repair. Biomaterials.

