# Supplementary material for: Fibrin glue as a stabilization strategy in peripheral nerve repair when using porous nerve guidance conduits
Source: J Mater Sci Mater Med. 2017 Apr 7;28(5):79. doi: 10.1007/s10856-017-5889-4 (PMC5384961; doi:10.1007/s10856-017-5889-4)
Supplement: Supplementary file 1 — Supplementary Table 1 [file 10856_2017_5889_MOESM1_ESM.docx]

**Supplementary Table 1**. Processing parameters and their effect on the HA and FG coating.

| HA(%w/v) | PEGDA(%w/v) | HA:PEGDA (mixed) | # of coatings | Observation |
| --- | --- | --- | --- | --- |
| 1 | 1 | 1:1 | Coated at gelation point- 1X | Pores open |
| 1 | 1 | 2:1 |  | Pores open |
| 1 | 1 | 4:1 |  | Pores open |
| HA(%) | PEGDA(%) | HA:PEGDA (alternate) | # of coatings | Observation |
| 1 | 1 | 1:1 | 1 | Pores open |
| 1 | 1 | 2:1 | 1 | Pores open |
| 1 | 1 | 4:1 | 1 | Pores open |
| 1 | 1 | 1:1 | 5 | Pores open |
| 1 | 1 | 2:1 | 5 | Pores open |
| 1 | 1 | 4:1 | 5 | Pores open |
| **1** | **1** | **1:1** | **20** | **Pores closed** |
| 1 | 1 | 4:1 | 20 | Pores open |
| Fibrin (mg/ml) | Thrombin (Units/ml) | F:T (mixed) | Observation |  |
| 5 | 20 | 4:1 | Pores open |  |
| 10 | 20 | 2:1 | Pores open |  |
| 16 | 1 | 1:1 | Pores open |  |
| 20 | 20 | 1:1 | Pores open |  |
| 20 | 20 | 2:1 | Pores open |  |
| 20 | 50 | 1:1 | Pores open |  |
| **50** | **50** | **1:1** | **Pores closed** |  |
| 100 | 50 | 1:1 | Pores open |  |
| 100 | 50 | 1:2 | Pores open |  |
| 100 | 50 | 1:4 | Pores open |  |
| 100 | 50 | 2:1 | Pores open |  |
| 100 | 50 | 4:1 | Pores open |  |

**Supplementary Fig. 1** Schematic process of the HA and FG coating on the porous braided nerve conduits.
