# Supplementary figures and images for: Fibrin glue as a stabilization strategy in peripheral nerve repair when using porous nerve guidance conduits
Source: J Mater Sci Mater Med. 2017 Apr 7;28(5):79. doi: 10.1007/s10856-017-5889-4 (PMC5384961; doi:10.1007/s10856-017-5889-4)

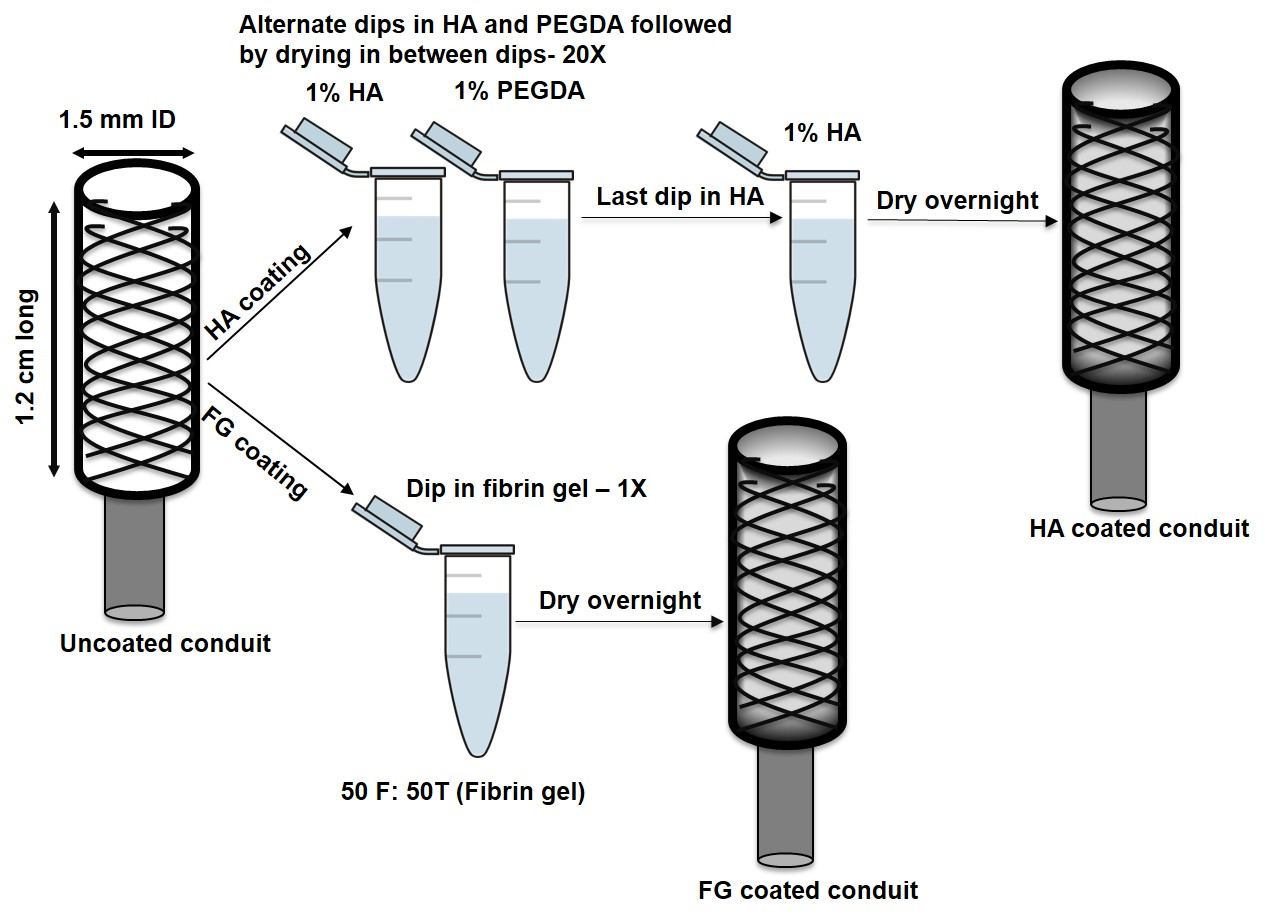

Supplement: Supplementary file 2 — Supplementary Fig. 1 [file 10856_2017_5889_MOESM2_ESM.tiff]
